# Sea ice surface temperatures from helicopter-borne thermal infrared imaging during the MOSAiC expedition

**DOI:** 10.1038/s41597-022-01461-9

**Published:** 2022-06-25

**Authors:** Linda Thielke, Marcus Huntemann, Stefan Hendricks, Arttu Jutila, Robert Ricker, Gunnar Spreen

**Affiliations:** 1 University of Bremen, Institute of Environmental Physics, Bremen, 28359 Germany; 2 Alfred Wegener Institute, Helmholtz Centre for Polar and Marine Research, Bremerhaven, 27570 Germany; 3 NORCE Norwegian Research Centre, Tromsø, 9019 Norway

**Keywords:** Cryospheric science, Ocean sciences

## Abstract

The sea ice surface temperature is important to understand the Arctic winter heat budget. We conducted 35 helicopter flights with an infrared camera in winter 2019/2020 during the Multidisciplinary Drifting Observatory for the Study of Arctic Climate (MOSAiC) expedition. The flights were performed from a local, 5 to 10 km scale up to a regional, 20 to 40 km scale. The infrared camera recorded thermal infrared brightness temperatures, which we converted to surface temperatures. More than 150000 images from all flights can be investigated individually. As an advanced data product, we created surface temperature maps for every flight with a 1 m resolution. We corrected image gradients, applied an ice drift correction, georeferenced all pixels, and corrected the surface temperature by its natural temporal drift, which results in time-fixed surface temperature maps for a consistent analysis of one flight. The temporal and spatial variability of sea ice characteristics is an important contribution to an increased understanding of the Arctic heat budget and, in particular, for the validation of satellite products.

## Abstract (summary)

**Table:** 

Measurement(s)	Surface temperature
Technology Type(s)	Infrared camera
Sample Characteristic - Environment	Sea Ice
Sample Characteristic - Location	Arctic Ocean

## Background & Summary

The measurement program was part of the Multidisciplinary Drifting Observatory for the Study of Arctic Climate (MOSAiC) expedition (https://mosaic-expedition.org), which took place in the Arctic from October 2019 to October 2020 and was divided into five legs^1^. RV Polarstern^2^ was drifting attached to an ice floe along the transpolar drift from about 85 N north of the Laptev Sea towards Fram Strait between Svalbard and Greenland^1,3^. To study the Arctic climate, a research camp, called Central Observatory (CO), was installed on a second-year ice floe that survived the summer and originated from the Laptev Sea^4^. The ice of the MOSAiC floe was very rotten and porous from the summer melt at the beginning of the drift. The level ice areas were characterized by a high fraction (>60%) of refrozen melt ponds, which had physical properties more similar to first-year ice. First-year ice was developing in the surroundings of the floe (later reaching about 30% fraction) and in cracks frequently cutting through the CO throughout the drift. Additional measurements were performed on the surrounding sea ice within a distributed network (DN) of autonomous measurement platforms and from the air by helicopters and drones.

From October 2019 to April 2020, we performed 35 helicopter flights with an Infrared camera (IR camera) on the Airbus Helicopter MBB-BK 117 C-1 over Arctic sea ice. All flights covered a similar sea ice area in the near vicinity of the drifting RV Polarstern. The IR camera took more than 150000 images in total with 640 × 48s0 brightness temperature values, which we converted to surface temperatures. All images of each flight are combined in one surface temperature map for every flight with a high spatial resolution of 1 m. The maps allow an investigation of spatial and temporal variability on a seasonal time scale based on the series of flights throughout the whole winter season. Measurements of the thermal radiation can be performed without daylight. Thus, thermal infrared observations are valuable source of data in the polar night during the Arctic winter. Detecting the thermal radiation is an appropriate tool because there are large temperature differences between open water or thin ice and snow-covered thick ice and helps to detect different surface characteristics which are, e.g., leads (cracks in the sea ice) as warm anomalies. Examples for the application of thermal infrared observations are lead detection^5^, sea ice concentration retrieval^6^, thin ice thickness estimation^7^, and surface heat flux estimates^8^.

Especially for the central Arctic, *in-situ* data rarely exist for the winter season. Therefore, operational observations are provided only by satellites. Here, our helicopter-borne, high spatial resolution data can resolve surface temperatures on a satellite sub-footprint scale which can help to improve the satellite retrievals. A high spatial resolution of 1 m is crucial since small-scale features like narrow cracks in the ice can release a significant amount of heat from the ocean to the atmosphere for the usual winter case when the atmosphere is colder than the water^9^. This becomes especially important in a warming Arctic^10^ while the sea ice is getting thinner, moves faster, and therefore breaks up more easily^11–14^. These small-scale cracks are not directly represented in thermal satellite data. Satellites are the main source for Arctic sea ice observation but have a spatial resolution of about 1 km in the TIR region. For example, Willmes and Heinemann (2016)^15^ developed pan-Arctic lead maps with a resolution of down to 1 km from data of the Moderate Resolution Imaging Spectroradiometer (MODIS). As they point out, the current uncertainties in satellite retrievals are, among other things, caused by missing validation datasets. Satellite-derived parameters are crucial for the evaluation of the larger regional and Arctic-wide climate context of the MOSAiC measurements like, e.g., the sea ice concentration, thickness, lead fraction, and ice drift time series along the MOSAiC drift track^3^.

As examples, we present and discuss three images and two maps from the IR camera observations to show the potential of the data. Beforehand, we explain the uncertainties and corrections, which have to be considered for the use of the data. We provide a detailed description of the processing method for the images, the mapping, and the approach to have a time-fixed surface temperature. We discuss the surface temperature distribution and the two-dimensional image- and map-based analysis for the investigation of the spatial distribution of specific features. Lastly, we give suggestions about possible applications for the IR images and maps.

## Methods

**Fig. 1:**
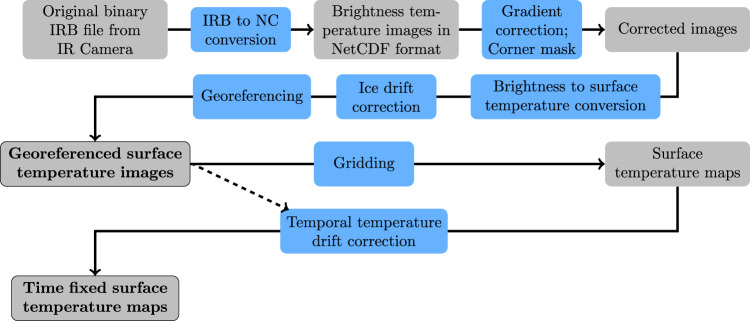
The flow chart is presenting the processing steps from the raw data of the IR camera to the time-fixed surface temperature map as part of the finished product (NC = NetCDF). The grey boxes indicate the data status and the grey, black-framed boxes with the bold font indicate the published data products. The blue boxes show the performed processing steps according to the methods explained in this study. The dashed arrow shows the input of the single images, which is used for finding the best fit of the temporal temperature drift correction.

The processing of the temperature images resulted in two types of datasets. First, the processed images are provided for every flight including the corrections applied here. Based on the corrected images, gridded data are provided as temperature maps which result from the whole set of images in one flight as an advanced dataset. The processing steps are displayed in Fig. 1. A list of flights and variables are described and, if necessary, updated in our data manual^16^.

### Camera details

**Fig. 2:**
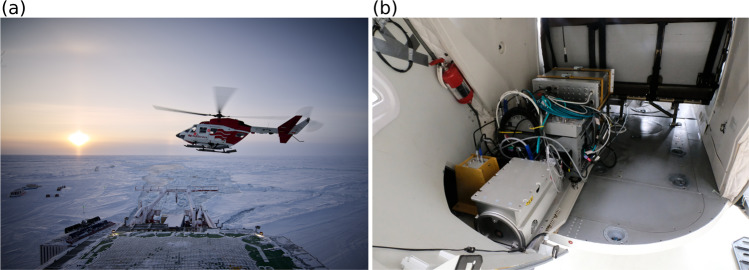
The images illustrate the measurement setup during the expedition. (**a**) The photo shows a helicopter flying close to RV Polarstern which is surrounded by sea ice (Credits: Alfred-Wegener-Institut/Michel Gutsche, CC-BY 4.0). (**b**) This photo shows the setup of the instruments in the cargo compartment of the helicopter. The IR camera is installed in the yellow box looking through a cutout (not visible) nadir downward towards the surface (Credits: Alfred-Wegener-Institut/Stefan Hendricks).

The images were recorded with the Infrared VarioCAM HD head 680 from InfraTec^17^ which was positioned in a helicopter with the nadir downwards view towards the sea ice surface (Fig. 2). All essential technical details about the IR camera are highlighted in this paragraph and listed in Table 1. The broadband thermal infrared imaging was performed in the thermal infrared region of the electromagnetic spectrum from 7.5 to 14 *μ*m, which includes the Earth’s radiation peak at 10 *μ*m [^18^, page 293]. The accuracy for the measured brightness temperature is 1 K and its precision is 0.02 K. To distinguish features on the ground within one image the precision is more important than the accuracy. The calibration of the device was done down to the target temperature of −8 °C with a measured deviation of 0.6 K. It has to be considered that the measurements had mainly been performed at lower temperatures. The minimum temperature by the manufacturer is −40 °C while we could measure even lower temperatures of the sky. During the measurement we reached the limit a few times, but did not exceed the limit extensively. Therefore the accuracy could slightly change for colder temperatures but this is neglected in this study. A sufficient overlap of the images is guaranteed by an acquisition rate of 1 Hz. The raw data were measured at an even higher frequency of 4 Hz, but down-sampled here to 1 Hz. During the recording, every 15 to 60 seconds a non-uniformity compensation (NUC) ensured that there was a minimized camera-internal drift in the temperature. The focus was adjusted after reaching the final flight altitude and stayed fixed for the whole flight. We assume to have the same focus for all flights because all measurements were performed at the similar flight altitude. More information about the device *ir_variocam_02* and its actions during the expedition are stored in the Alfred-Wegener-Institut (AWI) Sensor platform (https://sensor.awi.de/)^17^.

**Table 1:** Technical details for the Thermal IR camera VarioCAM HD head 680 GW from InfraTec GmbH Infrarotsensorik und Messtechnik.

Detector type	Uncooled microbolometer-FPA, Pitch 25 *μ*m
Detector format	(640 × 480) Pixel
Spectral range	(7.5… 14) *μ*m
Temperature range	(−40… 1200) °C
Thermal resolution	up to 0.02 K
Accuracy	1% for <0 °C
Field of view	(56.1 × 43.6)°
Focal length	15 mm
Focus	Manual

### Helicopter-borne survey flights

**Fig. 3:**
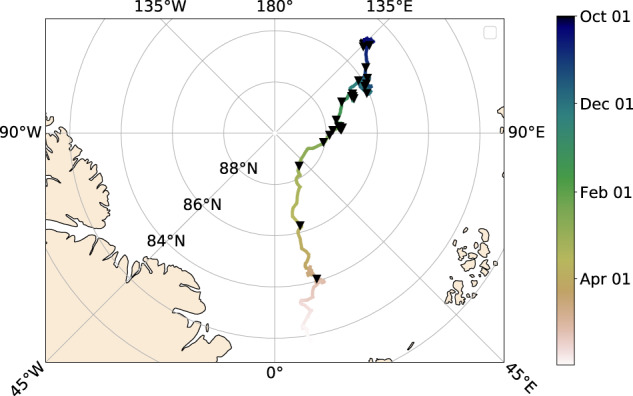
The map presents the locations of the measurements included in this study. The location of helicopter flights are marked with black triangles along the MOSAiC drift track from the northern Laptev Sea towards the Fram Strait with color-coded time.

The 35 flights were conducted during the first three legs of the expedition between 02 October 2019 and 23 April 2020 at an altitude of around 300 m and with a duration of about 90 minutes. The general objective of this measurement program was to have weekly flights. The frequency of flights varied because it depended on the weather conditions, was adapted to cover interesting events in the sea ice area, and some flights had to be aborted due to bad weather. There was a higher flight frequency in the earlier part of the drift, but a lower frequency in the latter part. This resulted in a larger geographic distance between the flights due to a faster ice drift during that period (Fig. 3); mind that the local ice surface did not necessarily change faster because of that, because the MOSAiC was drifting with the ice. Nine flights are not included in this dataset yet because they require additional processing, e.g., the IR camera has missing GPS data. They include seven flights from the wintertime and two flights during the last leg in September 2020 which were mainly conducted over thin ice areas at the ice margins and not cover the MOSAiC floe. Their data will be published at a later stage.

**Fig. 4:**
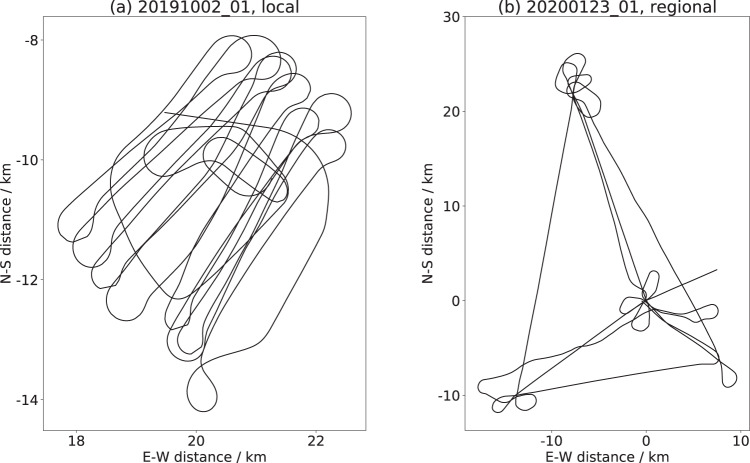
To get an idea about the coverage of surface for different flight patterns, we display the flight tracks for (**a**) local CO flight 20191002_01 and (**b**) regional L-site flight 20200123_01. The axes show the distance in meter. The center point (0,0) is the location of RV Polarstern. For the flight 20191002_01 in (**a**) (0,0) is outside the map because RV Polarstern was not located at the MOSAiC ice floe yet.

There are four different flight types in our measurement program that cover (i) the Central Observatory (CO), (ii) the three nodes of large sensor sites (L-Sites) (L1, L2, L3) in the Distributed Network (DN), (iii) single L-Site (grids), or (iv) a specific event, e.g., a lead opening. The CO flights (i) observed the local ice conditions of the MOSAiC ice floe in high spatial detail (Fig. 4(a)) and L-Site flights (ii) allowed an investigation of the ice conditions on a regional scale. The regional L-Site flights (ii) were flown in a triangle pattern covering the three L-Sites, located in the corners of the flight pattern (Fig. 4(b)). On 08 January 2020 the different L-Sites were overflown separately in the same patterns as the CO surveys, i.e. single L-Site grids (iii). The main weekly time series consists of the CO flights (i) and L-Site flights (ii).

### Sea ice surface temperature

In the thermal infrared range, the measured brightness temperature is close to the actual surface temperature. As a first approximation, the surface temperature *T*_*s*_ was calculated from the up-welling brightness temperature *T*_b,up_, measured with the IR Camera, and the emissivity *e* [^18^, page 294] with

1 $${T}_{{\rm{s}}}=\frac{{T}_{{\rm{b}},{\rm{up}}}}{e}.$$

For emissivities close to one, as in our case, the measured brightness temperature can be approximated to change linearly with the physical temperature. The Plank law is already considered during the recording of the brightness temperature. The influence of the down-welling brightness temperature was neglected for simplicity. The emissivity e is assumed to be a constant value of 0.996 for all incidence angles and surface types following Høyer *et al*. (2017)^19^. Here the downwelling contribution with (1-*e*) *T*_*s*_ would be 1 K at 250 K surface temperature which is the same range as the accuracy of the measured temperature. We used this emissivity, because the instrument of the Hoyer *et al*. study, i.e., a Cambell Scientific IR120 radiometer, has with 8 to 14 *μ*m a very similar spectral range to our instrument. However, the spectral weight function might differ. An emissivity of 0.97 was used for other infrared observations, e.g., by the NSIDC during Operation IceBridge flights with a KT19 thermometer with a spectral response from 9.6 to 11.5 *μm*. The lower emissivity value of 0.97 would lead to overestimated surface temperatures compared to our used value of 0.996. The accuracy of the surface temperature is determined by the accuracy of the brightness temperature (1 K, see Camera details) as well as of the accuracy of the emissivity. The additional uncertainty by the emissivity can be influenced by the incidence angle or change of surface type^20^.

In future studies, the aim is to apply a more detailed theory to retrieve a higher accuracy of the surface temperature *T*_*s*_. Additionally, it has to be considered that the penetration depth for thermal infrared radiation is very small of a few *μ*m, which allows an influence by atmospheric parameters, like the air temperature, wind speed, or cloud cover [^18^, page 294].

### Image correction

#### Corner mask

In all flights, warm temperature anomalies were found in one corner. The IR camera looked through an open cutout in the helicopter bottom structure. The higher values were probably caused by a shielding effect from the structure of the helicopter, which slightly influenced the temperature recording in the respective corner. To remove false high temperatures in the respective corner, a corner mask is provided for every flight and can be applied to each image. We generally recommend to exclude the corner areas of the images in any case due to distortions in the higher incidence angle regime.

#### Radial image gradient

**Fig. 5:**
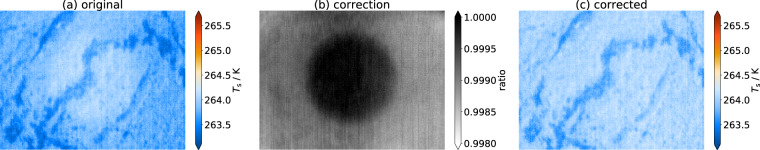
The image gradient correction of the surface temperature *T*_*s*_ is presented with an example image of the flight 20191002_01 with (**a**) the original image of surface temperature in K, (**b**) the correction array and (**c**) the corrected image of surface temperature in K.

In every image, a radial gradient of the recorded temperature occurs. This gradient is likely caused by an artificial effect of the camera. We corrected it by an empirical radial gradient which is calculated by averaging images of a flight and applied for every flight independently. For this, only images with an average temperature below the 25th percentile of all images are considered to exclude warm features like leads which could influence the resulting gradient. The gradient is relative to the center temperature, which is assumed to be the truth. This lens effect correction was applied to all datasets and the correction array is included in the image-based dataset for every flight so that the original, uncorrected images can be reproduced if needed. In Fig. 5, a case example from the flight on 02 October 2019 illustrates the correction of an image by the gradient. The original image (Fig. 5(a)) shows colder outer parts of the image. With the reference value in the center of the image, the image was corrected by a specific factor (Fig. 5(b)) resulting in a corrected image without a radial gradient (Fig. 5(c)).

### Mapping

**Fig. 6:**
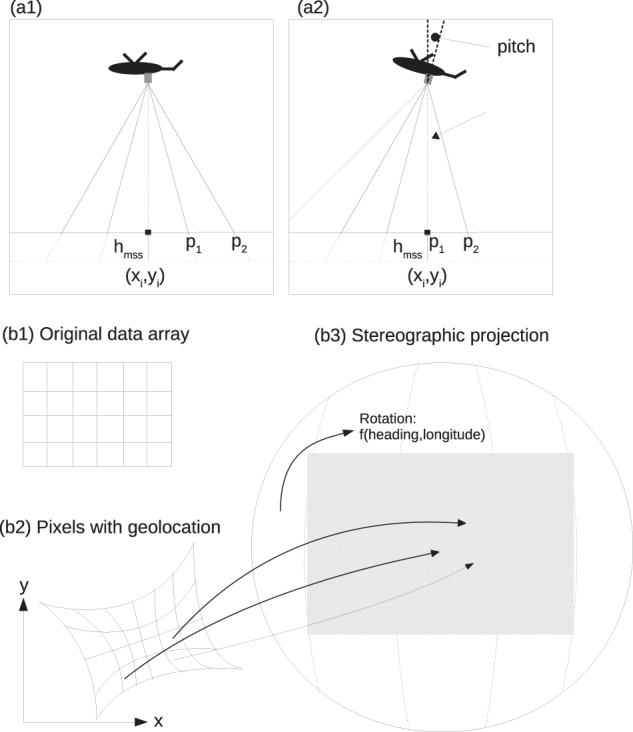
The schematic illustrates the different parts of the mapping method applied to the images. We calculate the positions on the ground based on the helicopter position (a1) which change with the helicopter position and rotation (a2). We also consider the reduction of the ellipsoid height from the GPS by the mean sea surface height *h*_*mss*_ because it changes the actual position of the pixel on the plane. The pixel locations are determined based on the reference point (*x*_*i*_, *y*_*i*_) at the known nadir location. In the next step, the projection method starts with the data array from the image (b1), which is further processed to the georeferenced images with x,y coordinates (b2). Finally, the pixels of the image are assigned to an equidistant grid with a stereographic projection (b3).

After processing the image data, we produced flight maps by combining all images in one grid. The gridded data set consists of temperature values with assigned coordinates (longitude and latitude) as well as relative coordinates to Polarstern for co-location with other measurements. This mapping was done in three steps, which are described in more detail in the following and which is illustrated in Fig. 6. First, the ice drift correction was performed to correct the coordinates to the target time which is the middle of the flight. Second, all image pixels had to be georeferenced under consideration of the helicopter position and rotation. Third and last, all pixels were assigned to the closest grid cell in the equidistant grid.

Some images were discarded if they were outside the main flight pattern, not yet at the main flight altitude level of around 300 m, or not usable due to the internal calibration (NUC). Here one image per NUC had to be discarded. As a benefit of excluding the discarded images, the maps are more compact in terms of data coverage of the covered area and better suited for the statistical and visual interpretation of the spatial variability in the defined area. In the following part, we explain the detailed methods for the image mapping.

#### Ice drift correction

Assuming the average ice drift of 8.52 km/d according to Krumpen *et al*.^3^, the sea ice drifted about 500 m during a 90-minute flight. Thus, a piece of ice at a specific location at the beginning of the flight was at another location later during the flight. Therefore, we corrected the coordinates of every image based on the drift of RV Polarstern to be in the coordinate system of the target time in the middle of each flight. The position and heading data^21–23^ of RV Polarstern, which was achored directly to the ice floe, were recorded with the high-performance inertial navigation system (INS) Motion Sensor Hydrins 1^24^. For the ice drift correction, we use linear interpolated data of 10-minutes interval Polarstern positions to correct each image accordingly with the assumption of homogeneous ice movement across the entire flight area. We took the GPS position at the target time as the reference point for the stereographic projection which is used for the final gridded temperature maps. RV Polarstern is always in the center of the stereographic projection and for the rotated relative coordinates RV Polarstern’s heading is defining the positive y-axis and the starboard direction is the positive x-axis. By this, relative distances of the flight data are kept comparable between all flights. The resulting map contains the locations of the observed sea ice surface at the target time. This was used to calculate a relative coordinate system with distances towards the reference point at RV Polarstern.

#### Georeferencing

Based on the corrected image location and the stereographic projection, every pixel is geolocated. All projections are done with the WGS84 ellipsoid. The geolocation of each pixel is determined based on a pinhole camera model and a transformation from the camera to the plane^25^. While the pitch angle was generally low during the flight, the roll angle was increased during turns and discarded in cases of a high angle with more than 40 degrees to avoid large distortions in the projection. Values from images with a high roll angle were discarded if data with a roll of less than 20 degrees were available. In general, we would recommend using the data of high roll angle with caution because large distortion can cause uncertainties in the geolocation. We correct the ellipsoid height, measured by the helicopter GNSS, by the mean sea surface height by Andersen and Knudsen (2009)^26^. Additionally, we perform a distortion correction for the images to reach a more accurate pixel position. We use the positioning data which were recorded with an embedded GNSS inertial system Applanix AP 60-AIR^27^ which was installed in the helicopter next to the IR camera (Fig. 2).

For an increased accuracy in the geolocation, we performed an optimization for the following six input parameters:

- Time (time offset between IR Camera and GNSS)
- Three components of a fixed camera rotation with respect to the helicopter (roll, pitch, heading)
- Internal camera parameters (effective focal length, radial distortion coefficient)

The optimization procedure consists of different steps. At first, common features between different images were determined using a consecutive execution of edge detection, pattern matching, homograhic fitting and cleaning using a RANSAC (RANdom SAmple Consensus^28^) based method. The euclidean distance between the same feature in pairs of two different images in the final coordinate system is subject to minimization. Since the feature detection, despite initial outlier correction, were not perfect, even for a good matching parameter, a high bias in the sum or mean of the determined distances. Therefore, we used a loss function employing the 70th percentile of the distances of ground projected point matches in one images pair and then another 70th percentile of these values from all used images as loss. This parameter was then minimized using an adaptive differential evolution optimizer^29^.

For our processing we determined two different sets of correction parameters because there was change of the camera setup at the start of Leg 2. The parameter are listed with the order (time offset, roll offset, pitch offset, heading offset, effective focal length, radial distortion coefficient):

- For Leg 1 (until 20191206_01): (0.49762, 0.458456, 0.134936, 88.3343, 604.362, −0.407934)
- For Leg 2 and 3 (20191224_01 onwards): (0.510041, 0.349084, −0.279992, 90.2153,604.107, −0.420221)

For the respective leg the optimization was done for one specific flight, i.e., 20191002_01 for Leg 1 and 20191224_01 for Leg 2. Since there was the same camera on all three legs, the focal length and radial distortion parameter should not change. As a result, determined by the optimization, these parameters ended up very close. For all flights, we use these optimized parameters. Additionally, we manually determined a time offset for each flight because the IR camera is recording the time only with the full second. But for a flight speed of around 45 m/s, a time difference of 0.5 s can already cause a significant position shift of the images. As expected, we have mostly a time correction below 1 s. In cases of a higher time shift, probably the required initial time synchronisation was not performed. The time offset for every flight is included in the image NetCDF file while the time variable contains the final (corrected) time used.

In addition, the following modifications had to be done during the processing:

- Until 20191130_01: rotation of image by 180° (camera was rotated in the helicopter)
- Excluding images in the middle of the flight 20191105_01 because of missing position data
- Correct heading by the difference of the actual longitude and the central longitude of the projection
- Change of sign of pitch angle

#### Gridding

After having a georeference for all pixels in every image, we assigned the pixels of all images to an equidistant grid of 1 m. The original images have a higher resolution of approximately 0.5 m at nadir. However, 1 m guarantees full coverage of all grid points for the final map, even under a higher incidence angle. After the ice drift correction, the pixel coordinates of all images are assigned to a grid cell of the stereographic grid map referenced to the target time (Fig. 6). In case several values fit in one grid cell, the value closest to the target time was taken and the overlap discarded. In addition to the gridded surface temperature, we included the longitude, latitude, time, roll, and pitch, measured by the Applanix AP 60-AIR^27^, as gridded data as well. This set of parameters contains all necessary information for the interpretation of the physical parameters. We also included relative coordinates which are comparable for the whole dataset period. They have one constant reference point at RV Polarstern whereas the longitude and latitude coordinates were changing constantly with the drift. The rotated relative coordinates have always the same orientation,also with RV Polarstern as reference (see Ice drift correction). The 5 m resolution are the block average of the 1 m resolution data.

#### Uncertainties of the geolocation

Uncertainties in the mapping can be caused by the ice drift correction which is well-defined for the area of the CO, close to RV Polarstern, but can differ for outer areas. The surrounding sea ice could have drifted differently than the CO during the flight, which cannot be considered because there are no data available. However, this effect is small for the duration of one flight. Three to five Automatic Identification System (AIS) base-stations were installed in the CO, which could have served as an alternative to provide the ice drift correction. However, compared to the ship GNSS and INS system the AIS stations showed jitter of tens of meters, i.e., noise in the GPS position with time which could lead to a temporal variability of the signal and therefore some inconsistencies. Therefore we decided to use the RV Polarstern position data. The only disadvantage of that would occur if RV Polarstern would move relative to the CO during a helicopter flight, which did not happen for our flights here mentioned. The uncertainty of the ship’s navigation system Hydrins is 0.01 deg for the heading and 0.03 m for the position^24^ which is neglectable for our 1 m resolution data.

Additionally, there are uncertainties caused by the installation of the instruments in the helicopter. The IR camera and the INS were deployed at different positions on the instruments plate in the helicopter, both with independent spring dampers (Fig. 2), which could respond differently to vibrations of the helicopter. This would result in small angular noise, which was neglected. The uncertainty from the positional difference is small and is mostly overcome with the optimization which provides us with the optimal input parameter for the given setup. The pixel distance at the surface is dependent on the flight altitude which we corrected by the mean sea surface height (Fig. 6(a1)), but did not consider the changes due to tides, currents, or atmospheric pressure loading. In addition, IR camera images were projected to a plane at the distance of the helicopter to the mean sea surface height, which is probably lower than the sea ice surface. But, compared to the difference to the actual surface height, we consider this error negligible. The outer areas of the images (100 pixels in y-direction, the small axis of the image) were cut off to reduce the effect of perspective and corresponding lowering of the resolution in the mapped data. Even with this cut of the data, coverage along flight direction is sufficient since the overlap is large. There might be a small time-drift in the IR camera time (<2 s/day, i.e., about 0.1 s per flight), which can reduce the matching of the position data and the image. Based on the results of the georeferencing and gridding, we estimate the error of geolocation to a maximum of 10 m. For the L-sites flights with large distances to RV Polarstern a larger shift is possible due to, e.g., inhomogeneous ice drift. Since the flights were performed quite far north it has to be considered that the INS data quality was degraded due to the close proximity to the earth rotational axes^30^. Further quantification of the actual pixel-wise uncertainty in geolocation can not be performed with the methods available to us. Small shifts in the geolocation can cause issues in the matching of the same features from different images, but this does not change the statistics of the surface temperatures. Further quantification of geolocation uncertainty requires co-location with other point measurements on the floe which was not performed for this dataset.

### Temperature-drift correction

During most flights, the temperature changes with time. This becomes clearly visible when different swathes in one flight are crossing. As an example, two neighboring images from different times within a flight have a temperature difference of 2.6 K for the local flights 20191105_01 or 20191230_01 and even 3.95 K for the regional flight 20191230_01. That is physically reasonable because changes in atmospheric parameters like cloud cover, wind speed, or air temperature can alter the surface temperature within the flight duration of about 90 minutes. A further investigation of the reasoning for this temperature drift will be performed in another study. However, this temporal temperature change would make it challenging or not possible to analyze the temperature maps of a whole flight coherently. Thus, we derived the time-fixed surface temperature by a temporal correction of the temperature change. This makes surface features in the data more easily interpretable because the temporal temperature changes do not have to be taken into account. For this, we corrected the surface temperatures of a whole flight by relative differences to the target time in the middle of the flight. The fixed surface temperature *T*_*s,fixed*_ was corrected based on the fit function *f*_*i*_ of the 10th percentile as lowest surface temperature *T*_*s*_ values for each image (Eq. 2). The 10th percentile was chosen because it represents the fairly constant background temperature of cold snow-covered thick ice and is not influenced by varying warmer features like cracks in the sea ice.

The temporal temperature change was corrected by a fit function with the target time *t*_0_ and the actual time of the pixel *t* (Eq. 2):

2 $${T}_{s,fixed}={T}_{{\rm{s}}}-({f}_{i}(t)-{f}_{i}({t}_{0})),$$

whereas *f*_*i*_ represents the fit function. Since there are different temporal behaviour for the flights, there are four fit types applied on every flight which are tested for all flights: 1) linear, 2) quadratic, 3) third-degree polynomial, and 4) exponential (Eq. 3 to Eq. 6):

3 $${f}_{{\rm{lin}}}(t)={{\rm{a}}}_{1}\cdot t+{\rm{c}}$$

4 $${f}_{{\rm{quad}}}(t)={{\rm{a}}}_{2}\cdot {t}^{2}+{{\rm{a}}}_{1}\cdot t+{\rm{c}}$$

5 $${f}_{{\rm{poly}}}(t)={{\rm{a}}}_{3}\cdot {t}^{3}+{{\rm{a}}}_{2}\cdot {t}^{2}+{{\rm{a}}}_{1}\cdot t+{\rm{c}}$$

6 $${f}_{{\rm{\exp }}}(t)={\rm{a}}\cdot {\rm{\exp }}\left(-{\rm{b}}\cdot \left(t-{t}_{0}\right)\right)+{\rm{c}}.$$

The best fit type was selected by the smallest Chi-squared statistic. The selected, flight-specific fit type and parameters are listed in Table 2. The fit parameters a_1_, a_2_, a_3_, a, b, and c are fixed for each flight. The time *t* is given in seconds since day start. For the exponential fit, the initial guess was: (a = 0, b = 0.0001, c = 200). Some flights needed a slightly different initial guess with (a = −0.1, b = 0.0001, c = 200) or (a = 0, b = −0.0001,c = 200) because otherwise no solution could be found. The fit was performed to data only in the vicinity of the center of the CO, i.e. the position of RV Polarstern with 1 km extent in every direction and 100 measurement points from the start and end of the flight to cover the whole period. Often the flight starts and ends close to RV Polarstern anyway. As an exception, the mean longitude and latitude were used as the center point for the first CO and the three L-Site grids because during the flight RV Polarstern was not overflown. This restriction ensures that mostly the same sea ice surfaces of the CO are considered for the fit. That is especially important for the regional flights where a larger area and therefore a larger variability of surface temperatures is included. The temperature range for every flight (Table 2) is an indicator of the magnitude of temperature change in the respective flight. Here we considered the range between start and endpoint as an estimation of the homogeneity of the respective flight.

**Table 2:** Temporal temperature correction: Fit parameters of surface temperature *T*_*s*_ according to Eqs. 3, 4, 5, and 6 for every flight.

Flight ID	Fit type	a1	a2	a3	c	a	b	T_start_/K	T_end_/K	*T*_range_/K
20191002_01	linear	−0.000072	—	—	266.302890	—	—	264.74	264.58	−0.16
20191020_01	exponential	—	—	—	258.045648	−4.302381e + 00	1.501635e-03	253.74	258.00	4.26
20191029_01	linear	0.000054	—	—	255.915111	—	—	256.40	256.57	0.17
20191105_01	polynomial	−0.409536	1.584149e-05	−2.044194e-10	3774.198780	—	—	245.71	241.73	−3.98
20191112_01	exponential	—	—	—	248.708455	−4.325600e-03	−1.497353e-03	248.70	246.61	−2.09
20191112_02	linear	−0.000245	—	—	256.629279	—	—	247.42	246.94	−0.49
20191119_01	polynomial	−0.598430	3.687198e-05	−7.537312e-10	3475.193411	—	—	252.54	255.19	2.65
20191130_01	exponential	—	—	—	233.891970	1.797889e + 00	2.399643e-03	235.69	233.89	−1.80
20191206_01	exponential	—	—	—	239.973808	−6.599342e-01	1.239892e-02	239.31	239.97	0.66
20191224_01	exponential	—	—	—	239.124792	−2.166376e-01	−3.849560e-04	238.91	236.91	−1.99
20191225_01	linear	−0.000383	—	—	259.256967	—	—	239.58	237.66	−1.92
20191228_01	linear	−0.000114	—	—	235.795076	—	—	232.89	232.39	−0.50
20191230_01	exponential	—	—	—	237.346076	4.226815e + 00	2.073296e-03	241.57	237.35	−4.23
20200107_01	exponential	—	—	—	234.376458	8.064973e-07	−2.406496e-03	234.38	234.79	0.41
20200107_02	linear	0.000234	—	—	223.698382	—	—	234.05	235.34	1.29
20200108_01	exponential	—	—	—	3938.795777	−3.699968e + 03	4.535519e-08	238.83	239.20	0.38
20200108_03	linear	0.000770	—	—	213.448575	—	—	240.43	242.26	1.83
20200108_04	polynomial	6.814246	−1.428722e-04	9.981858e-10	−108058.120312	—	—	240.30	238.96	−1.34
20200116_01	exponential	—	—	—	240.280738	2.140605e + 00	7.520096e-04	242.42	240.36	−2.06
20200116_02	exponential	—	—	—	240.638773	−1.312025e + 00	5.731116e-04	239.33	240.58	1.25
20200121_01	exponential	—	—	—	233.777378	2.341788e + 00	2.278946e-03	236.12	233.78	−2.34
20200123_01	exponential	—	—	—	233.528294	3.893223e-01	2.898193e-03	233.92	233.53	−0.39
20200123_02	linear	0.000039	—	—	231.193934	—	—	233.08	233.20	0.12
20200125_01	quadratic	−0.001041	3.797253e-09	—	274.179663	—	—	234.41	233.27	−1.14
20200128_01	polynomial	−0.060552	2.275804e-06	−2.846043e-11	766.664327	—	—	230.98	230.40	−0.58
20200202_01	quadratic	−0.001395	1.891434e-08	—	254.646072	—	—	231.93	231.36	−0.57
20200204_01	quadratic	−0.010815	1.760454e-07	—	402.498456	—	—	239.63	237.34	−2.29
20200209_01	linear	−0.001180	—	—	286.716503	—	—	239.57	233.12	−6.45
20200212_01	polynomial	−0.013750	3.870068e-07	−3.623340e-12	394.977441	—	—	232.53	232.48	−0.04
20200217_01	polynomial	−2.414028	9.524740e-05	−1.252518e-09	20624.648178	—	—	233.12	232.82	−0.31
20200217_02	exponential	—	—	—	232.575541	−7.909334e-01	1.417581e-03	231.78	232.58	0.79
20200227_01	quadratic	−0.004935	5.481852e-08	—	343.906063	—	—	233.00	232.92	−0.08
20200321_01	quadratic	−0.000908	1.255406e-08	—	250.002788	—	—	234.13	233.57	−0.56
20200321_02	linear	−0.000634	—	—	261.227104	—	—	237.33	234.14	−3.19
20200423_01	exponential	—	—	—	248.087844	2.106613e + 00	−9.915066e-05	250.19	252.07	1.88

For each flight the best fit type (linear, quadratic, polynomial, or exponential) is selected. The start and end temperature (*T*_start_ and *T*_end_) as well as the resulting temperature range *T*_*range*_ indicate the trend and the amount of temperature change within a specific flight.

## Data Records

All data of the IR camera, including the images, the maps, and the plots of the maps, are published in PANGAEA with open access^31^. Specific information about the data, like the list of processed and unprocessed flights as well as definitions of variables and their dimensions can be found in our data manual^16^, which will be updated if changes occur. The data were converted from the raw, binary format IRB to a NetCDF4 file format. There are two types of datasets with one that contains the image-based data and the other that contains processed maps with 1 m and 5 m horizontal resolution. The 5 m grid is added for smaller file size and easier data handling but is otherwise identical to the 1 m dataset. Each dataset type contains one NetCDF file for each flight. For the single images dataset all images of one flight are combined in one NetCDF file with the single images aligned along the third dimension (time). All files include the stereographic projection parameters used for the mapping. The flights have two identifications that can be used: (i) the Flight-ID, including the date of the flight, and (ii) the Device Operation, a unique ID, which was created during the MOSAiC expedition. The main variable is the surface temperature complemented by additional data necessary to process and interpret the data. All the position data of the helicopter are required to reference each pixel to the correct geolocation. The position data are published with the temperature data from the IR Camera but recorded with the embedded GNSS inertial system Applanix^27^ which was installed in the helicopter as well. The position data of RV Polarstern^21–23^ from the device Hydrins 1 are used in a temporal resolution of 10 minutes, but were interpolated to 1 second for the ice drift correction. Also the 10 min weather data from Polarstern are used^32–34^. Both, position and weather data, were downloaded from the AWI data platform (https://dship.awi.de). The mean sea surface height data is based on Andersen and Knudsen (2009)^26^ and taken from https://ftp.space.dtu.dk/pub/DTU21/1_MIN/. The processing steps of the data are summarized in Fig. 1.

## Technical Validation

### Temperature structures in images

Single images have a lot of information and illustrate the potential of the data. Here we present three example images from 05 November 2019, 25 January 2020, and 23 April 2020. Please mind that the three cases have different temperature ranges.

**Fig. 7:**
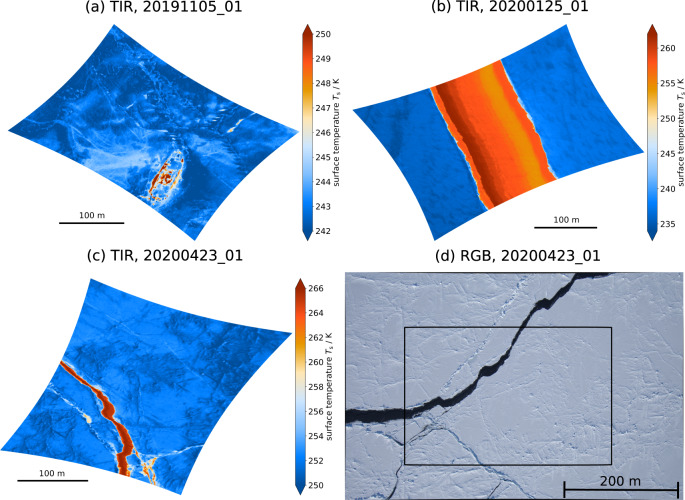
The images show example images recorded with the IR camera. Please note that all TIR images have a different temperature range and are oriented with North to the top and East to the right. (**a**) Surface temperature image from the flight 20191105_01 at 07:22:14 UTC. The warm structures show RV Polarstern in the lower part. Next to the ship, the human influence is visible by slightly warmer surface temperatures in the logistics area and the tracks going away from the ship. Topographic features stand out from a more homogeneous area in the upper part of the image. (**b**) Surface temperature image from the flight 20200125_01 at 12:49:11 UTC. The warm middle part is a crack in the ice with different warm temperature regimes (reddish) indicating different thin ice thicknesses. The outer sides are colder (bluish) because of thick ice which is covered by snow. (**c**) Surface temperature image from the flight 20200423_01 at 07:45:47 UTC. The area is dominated by cold temperatures due to thick, snow-covered, and partly deformed ice. A warm crack is reaching from the lower part to the middle left part. In the lower right part of the image, smaller cracks are visible. (**d**) The RGB image form 23 April 2020 is taken at the same time as the TIR image (**c**) and shows the surface characteristics in the visible range. The black rectangle define the area covered by the TIR image (**c**). The thick, snow-covered sea ice surface appears white while the crack is dark due to the ocean color. (Credits for the RGB image: Alfred-Wegener-Institut/Gerit Birnbaum).

Figure 7(a) illustrates the thermal signature due to human influence. RV Polarstern is visible in the image due to the warm structures of the ship. A warmer area on the starboard side of the ship was the logistics area and appears because some snow was compressed or removed and therefore the heat conductivity increased. Even single tracks can be identified because they are warmer than the surroundings which can be explained with the same principle of heat conduction. Other features in the bottom half of the image are linear and cold structures, which indicate the deformation of the ice, i.e., ridges. We can also identify blurry, cold spots that could be caused by snowdrift. Thus, topographical features can be investigated from this two-dimensional temperature variation.

In Fig. 7(b), a lead with internal surface temperature variability is present. The warmer temperatures in the middle of the image are caused by a newly opened lead, which is covered by thin ice, whereas the surrounding thick ice is substantially colder. There is a temperature change orthogonal to the lead edges with warmer temperatures on the left-hand side and colder on the right-hand side. The temperature difference can be explained by different thin ice thicknesses which could be caused by ice movement and associated rafting due to wind forces. Wind pressure can cause compressed and therefore colder ice whereas reduced ice thickness leads to higher temperatures.

For Fig. 7(c) most of the surface is thick, snow-covered ice, which is characterized by colder temperatures. The warm linear structure, i.e., a lead, reaches from the lower left to the upper middle part. Next to the lead, there is a slightly warmer line and in the lower left a high variability of warm and cold surface temperatures. The corresponding RGB image, taken at the same time, illustrates the visual appearance of the same surface (Fig. 7(d)). We selected this particular case because sufficient daylight was available for the RGB images, whereas during the polar night it was too dark to see the surface in the visible range. The white area corresponds with the cold, snow-covered sea ice, whereas the warm crack has the dark ocean color. The comparison illustrates that the surface of leads is affected predominantly by the warmer ocean compared to the colder atmosphere. Also, the thin line next to the lead can be identified in the visible range. The high variability in the lower left part is caused by broken-up ice, i.e., small floes, which result in partly missing insulation from consolidated snow-covered ice.

### High resolution temperature maps

Combining the images to a map provides the opportunity to analyze the spatial characteristics and variability of the surface temperature for a larger area. The gridded temperature data are corrected for ice drift as well as for the temporal temperature change as explained above and named as time-fixed surface temperature.

**Fig. 8:**
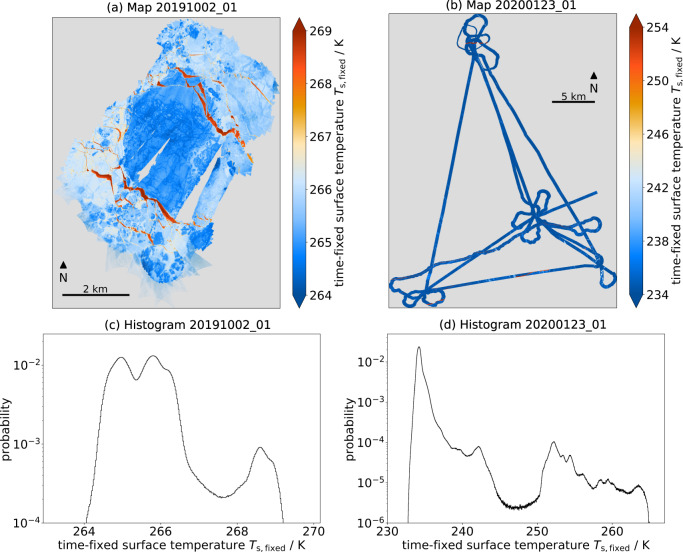
The combination of maps (**a,b**) and histograms (**c,d**) contains the surface temperature information of the advanced data product. Please note the different temperature ranges for the two flights. (**a**) The time-fixed surface temperature map of the local CO flight 20191002_01 is dominated by cold temperatures (bluish) in the middle part where there is thicker ice surrounded by thinner ice (warmer, light blue). Warm linear structures (reddish) are cracks in the ice, created by ice dynamics, with a surface cover of open water or thin ice. (**b**) The time-fixed surface temperature map of the regional L-Site flight 20200123_01 consists of mainly cold temperatures (bluish) representing thick, consolidated ice broken by a few cracks (reddish) in the southernmost east-west line and the northernmost L-Site. The black diamond indicates the position of RV Polarstern whereas for 20191002_01 RV Polarstern was not at the floe yet. The logarithmic probability of the time-fixed surface temperature for the gridded data for (**c**) local CO flight 20191002_01 and (**d**) regional L-Site flight 20200123_01. With the logarithmic scale, the small probabilities towards the higher temperatures are better distinguishable. The main maxima in the respective cold range indicate the main ice classes.

Figure 8(a) shows a map of the time-fixed surface temperature for the local flight on 02 October 2019 and shows the initial MOSAiC ice floe and its surroundings. This flight covered the area of and around the CO which was not installed yet. In the center, very low temperatures can be associated with thicker ice which survived the summer and was the main area for the CO. The surroundings have higher temperatures which represents thinner ice. The central thicker ice area is framed by warm thin structures from the north-east and south-west. These are cracks in the ice with a surface of open water or thin ice.

The time-fixed surface temperatures for the regional flight on 23 January 2020 appear cold and rather homogeneous (Fig. 8(b)). This is plausible because the air temperature was very cold for a longer period. Consequently, the ice thickness increased and the MOSAiC ice floe consolidated with the surrounding ice. For this triangle flight pattern, the maps are more difficult to interpret because the area is larger with a smaller coverage of surface temperature data. Nevertheless, warmer linear structures, i.e. leads, are present in this flight, for example at the northernmost L-Site as well as between the two southern L-Sites.

The temperature distributions of the two flights have different characteristic (Fig. 8(c,d)). The flights have a different temperature regime and a different range (1st to 99th percentile). The local flight on 02 October 2019 reaches from 264.42 K to 268.77 K, resulting in a range of 4.35 K, whereas the regional flight on 23 January 2020 has a wider range from 233.56 K to 252.51 K, resulting in a range of 18.96 K. Local minima discriminate different ice classes or surface types.

The local flight (Fig. 8(c)) has two peaks in the colder part and one at the warmer end of the temperature distribution. These peaks in the distribution reflect the sea ice properties described above. The warmest peak at 268.6 K represents the cracks in the ice covered by open water or thin ice. The coldest peak at 265.0 K is caused by the thick ice of the future CO in the center of the map whereas the middle peak at 265.8 K is related to the slightly thinner ice in the surroundings.

The regional flight (Fig. 8(d)) has mainly very low surface temperatures and therefore the global maximum in the coldest part of the distribution at 234.2 K. Aligned with the two-dimensional appearance in the map, the ice conditions on 23 January 2020 were dominated by thicker ice. Still, there is a variation in the warmer part of surface temperature distribution, associated with thin ice, which is separated from the lowest temperatures by the minimum at around 247.5 K. This thin ice cover can still appear under very cold conditions because it started to grow later in cracks of the ice that were opened due to ice dynamics. The several local minima in the warmer part of the distribution indicate that there are several distinct thin ice classes. These are likely associated with lead opening events at different lag times before the overflight, i.e. the length of the ice growth period is different for the different temperature classes. Another option is rafting of thin ice in leads.

### Artefacts in the maps

**Fig. 9:**
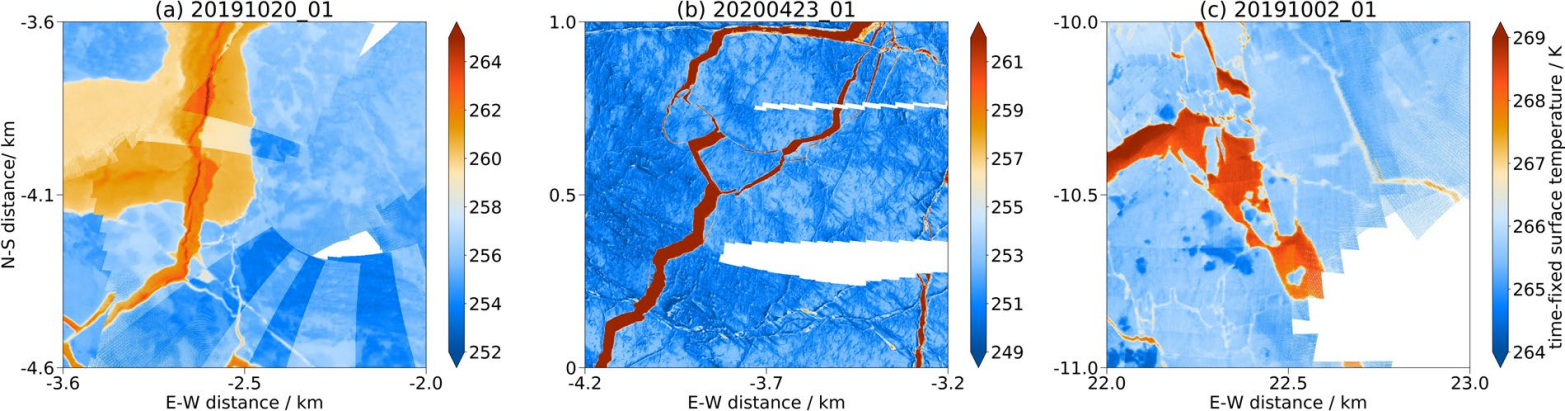
Three examples of artefacts from three different flights which can occur in the surface temperature maps. All examples show a 1 km by 1 km part of the 1 m resolution map. In panel (**a**) temperature jumps from flight 20191020_01 are shown. They occur because images from different times are taken while the surface temperature has a temporal drift during the flight. In panel (**b**) a geolocation offset between different swaths of flight 20200423_01 is illustrated, probably caused by irregular ice drift in between the recording. Panel (**c**) shows blurry structures in a area with high incidence angles during the flight 20191002_01.

As already discussed in the methodology there are some uncertainties with the data, which influences the outcome of our processing, i.e, the maps. In Fig. 9, we present possible artefacts the user should be aware of. Within the surface temperature maps temperature jumps can exist (Fig. 9(a)). There are two possible reasons. 1) The regular internal calibration adjusts the temperature to compensate for a temperature drift and thus can cause an abrupt shift. 2) (shown here) The temporal drift which could not be fully corrected results in a temperature shift along the intersection of two swaths with a given time difference. The geolocation uncertainty (Fig. 9(b)) can cause shifts between two overlying tracks which results in a discontinuity of the sea ice structures. Figure 9(c) shows the influence of high incidence angles. Large incidence angles (i.e., roll) during a turn have larger distortions and can have slight shifts in geolocation. From this, blurry structures can be caused and therefore we recommend using temperature values with incidence angles (roll) smaller than 20 degrees. But for statistical interpretation, the high incidence areas can be used as additional data. For a low temperature contrast scene, vertical lines can be visible. These vertical lines come from the images and are a known issues of the measurement technology of microbolometers as in Alhussein and Haider (2016)^35^.

### Atmospheric conditions

**Fig. 10:**
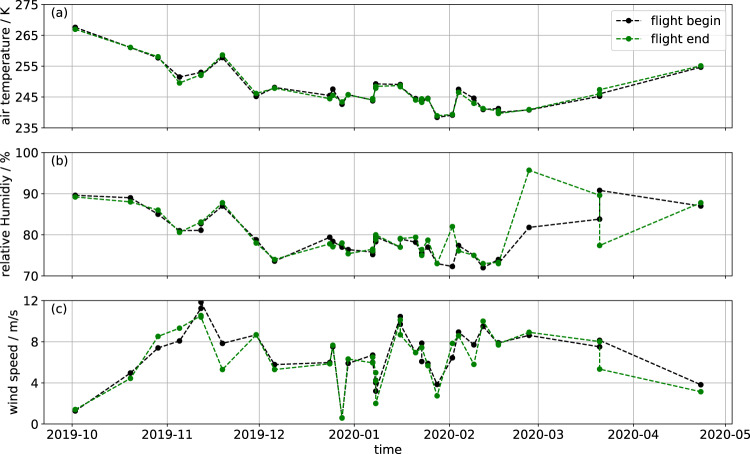
The meteorological conditions at Polarstern^32–34^ at the beginning (black) and end (green) of all flights for the parameter (**a**) air temperature, (**b**) relative humidity, and (**c**) wind speed. Additional cloud information are listed in Table 3.

As described above, the surface temperature is influenced by the atmosphere. Here we provide the basic meteorological information for all flights. There is no sufficient data for a quantitative analysis. The atmospheric data lack the spatial information which we would need to cover our flight area, especially for the spatially variable cloud coverage. The atmospheric parameters from Polarstern are presented with start/end conditions in Fig. 10 and the cloud conditions from the flight weather reports^36^ are listed in Table 3. This helps to better assess the potential atmospheric effect on the surface temperature. The flights were performed only during calm conditions which reduce the atmospheric influence to a minimum. The atmospheric parameters measured at Polarstern are mostly stable within one flight while they only change from flight to flight (Fig. 10). The air temperature (Fig. 10(a)) was stable within each flight with changes of at most 2 K. The relative humidity changes (Fig. 10(b)) for the flights on 27 February 2020 and 21 March 2020 and thus can influence the measured surface temperature due to changes in emissivity within the atmospheric pathway. Figure 10(c) shows that the wind speed varies the most within the flights in October/November and on 21 March 2020. The given cloud conditions are expected to have a minor influence except for potential local fog patches or sea fog over leads. The reported clouds are considerably higher than the flight altitude. Nevertheless, a change in cloud cover could change the incoming longwave radiation and therefore the surface temperature.

**Table 3:** Cloud conditions during the helicopter flights based on the flight report by the DWD^36^.

Flight ID	Cloud coverage	Cloud type	Lower cloud level/ft	Low clouds/mist/fog
20191002_01	BKN/OVC	ST/SC	800–1300	possible
20191020_01	SCT/BKN	SC	2000–3000	possible
20191029_01	BKN/OVC	SC	3000–4000	possible
20191105_01	SCT/BKN	SC/AS	3000–4500	occasional
20191112_01	SCT/BKN	SC/AS	3000–4000	occasional
20191112_02	SCT/BKN	SC/AS	3000–4000	occasional
20191119_01	SCT/BKN	CI/CS	>10000	occasional
20191130_01	—	—	—	occasional
20191206_01	FEW/SCT	AC/CI	>10000	occasional
20191224_01	—	—	—	occasional
20191225_01	SCT	SC	2000	occasional
20191228_01	SCT/BKN	SC/AC	2500–4500	possible
20191230_01	BKN	SC/AC	4000–5000	possible
20200107_01	SCT/BKN (isolated)	SC	1500–3000	possible
20200107_02	SCT/BKN (isolated)	SC	1500–3000	possible
20200108_01	SCT/BKN (isolated)	SC	4000	possible
20200108_03	SCT/BKN (isolated)	SC	4000	possible
20200108_04	FEW/SCT (isolated)	SC	3500–6000	possible
20200116_01	SCT/BKN	ST/SC	400–1000	occasional
20200116_02	SCT/BKN	ST/SC	400–1000	occasional
20200121_01	SCT/BKN (isolated)	SC	2500–3500	possible
20200123_01	FEW/SCT	ST	200–400	possible
20200123_02	FEW/SCT	ST	200–400	possible
20200125_01	FEW/SCT	ST	300	possible
20200128_01	SCT/BKN (isolated)	ST	300–600	possible
20200202_01	SCT/BKN (isolated)	ST	200–600	possible
20200204_01	SCT/BKN (isolated)	ST	200–600	possible
20200209_01	SCT/BKN	AS	>6000	possible
20200212_01	SCT/BKN (isolated)	ST	500	possible
20200217_01	SCT/BKN (isolated)	ST/SC	500–1000	possible
20200217_02	SCT/BKN (isolated)	ST/SC	500–1000	possible
20200227_01	SCT/BKN (isolated)	ST	500	possible
20200321_01	SCT/BKN (isolated)	ST	1000	possible
20200321_02	SCT/BKN (isolated)	ST	1000	possible
20200423_01	FEW/SCT (isolated)	AC	8000	possible

Please note, that high cirrus are not included if low clouds are present, because the low clouds have a significant higher influence on the surface temperature. The used abbreviations for cloud type are: AC = Altocumulus, AS = Altostratus, CI = Cirrus, CU = Cumulus, SC = Stratocumulus, ST = Stratus; and for cloud coverage: FEW = Few (1/8–2/8), SCT = Scattered (3/8–4/8), BKN = Broken (5/8–7/8), OVC = Overcast (8/8).

## Usage Notes

All data are provided in a NetCDF and do not need specific software to be read.

### Images

The image-based data include time, positioning of the device, and georeferenced coordinates for each pixel which allows gridding of all images without repeating the georeferencing. Thereby the overlap of the images can be used differently than done here or other methods could be applied for the gridding. Based on the temperature arrays and the position and rotation data of the helicopter, the georeferencing can be reproduced.

### Maps

The map-based data are provided with longitude/latitude coordinates as well as the relative coordinates, which are distances relative to the position of RV Polarstern. The rotated relative coordinates provide a consistent coordinate system for all flights. This allows co-locating measurements for different times during the expedition while the longitude and latitude are changing due to the ice drift. For co-location with measurements without direct relation to the expedition, longitude and latitude can be used. For the analysis of the time series of gridded data, the RV Polarstern-centered coordinate system based on a stereographic projection with constant relative distances is recommended. Within the range of 5 to 40 km, the distortion is assumed to be negligible.

### Application examples

Further ideas for the application of the surface temperature could be the use of temperature distributions of the sea ice surface to derive other surface properties, like ice types. Two-dimensional temperature patterns can be used to determine topographic features, which is difficult based on the one-dimensional temperature distribution due to the small temperature differences. Besides the statistical analysis, digital image processing methods could be applied to the image data. Generally, there is a lot of potential for analysis and comparison, in particular, in the context of the broad spectrum of measurements during the MOSAiC expedition^37–39^. The variety of measurements taken during the expedition should motivate to compare findings among different variables to gain an improved understanding of connected processes between ocean, ice, air as well as biochemical components of the Arctic. Based on the data presented here, further investigations of the thermal properties of sea ice will be performed and compared to other measurements taken at the same time and/or within the same area to gain a better process understanding of the Arctic climate system with the focus on the snow/ice-air interface. While the dataset presented here is suitable for most applications, there is still room for improvement for specific cases, mainly in terms of accuracy for the surface temperature values and the geolocation. All presented data are available under open access. New approaches for the data are encouraged and discussions with the authors of this paper are welcome.

## Data Availability

The data processing and analysis were performed with Python 3. The code responsible for processing and analyzing the maps and the images is published under open access^40^. The used colormap for the temperature is individually defined and provided as well. It is suited to visualize temperature structures for Arctic winter conditions. In case of specific requests, please contact the corresponding author directly.

## References

[1] Shupe MD (2020). NOAA.

[2] Alfred-Wegener-Institut Helmholtz-Zentrum für Polar- und Meeresforschung. Polar Research and Supply Vessel POLARSTERN operated by the Alfred-Wegener-Institute. *Journal of large-scale research facilities* **3**, 10.17815/jlsrf-3-163 (2017).

[3] Krumpen T (2021). MOSAiC drift expedition from October 2019 to July 2020: sea ice conditions from space and comparison with previous years. The Cryosphere.

[4] Krumpen T (2020). The MOSAiC ice floe: sediment-laden survivor from the Siberian shelf. The Cryosphere.

[5] Willmes S, Heinemann G (2015). Pan-arctic lead detection from modis thermal infrared imagery. Annals of Glaciology.

[6] Ludwig V, Spreen G, Pedersen LT (2020). Evaluation of a new merged sea-ice concentration dataset at 1 km resolution from thermal infrared and passive microwave satellite data in the arctic. Remote Sensing.

[7] Adams S (2012). Improvement and sensitivity analysis of thermal thin-ice thickness retrievals. IEEE Transactions on Geoscience and Remote Sensing.

[8] Qu M (2019). Estimation of turbulent heat flux over leads using satellite thermal images. The Cryosphere.

[9] Maykut GA (1978). Energy exchange over young sea ice in the central Arctic. Journal of Geophysical Research: Oceans.

[10] Dai A, Luo D, Song M, Liu J (2019). Arctic amplification is caused by sea-ice loss under increasing CO_2_. Nature communications.

[11] Kwok R (2018). Arctic sea ice thickness, volume, and multiyear ice coverage: losses and coupled variability (1958–2018). Environmental Research Letters.

[12] Spreen G, Kwok R, Menemenlis D (2011). Trends in Arctic sea ice drift and role of wind forcing: 1992-2009. Geophys. Res. Lett..

[13] Meredith, M. *et al*. Polar Regions. Chapter 3, IPCC Special Report on the Ocean and Cryosphere in a Changing Climate. *IPPC* (2019).

[14] Stroeve JC (2012). The Arctic’s rapidly shrinking sea ice cover: a research synthesis. Climatic change.

[15] Willmes S, Heinemann G (2016). Sea-ice wintertime lead frequencies and regional characteristics in the Arctic, 2003–2015. Remote Sensing.

[16] Thielke L (2022). Zenodo.

[17] SENSOR. Device Information: Infratec VarioCAM HD head 680. *AWI Sensor Web* https://hdl.handle.net/10013/sensor.0542bbfb-172a-496f-9bce-b21c59cd02c9 (2021).

[18] Shokr, M. & Sinha, N. *Sea ice: physics and remote sensing* (John Wiley & Sons, 2015).

[19] Høyer, J. L. *et al*. Report from Field Inter-Comparison Experiment (FICE) for ice surface temperature. *Danish Meteorological Institute* (2017).

[20] Hori M (2013). Modeling angular-dependent spectral emissivity of snow and ice in the thermal infrared atmospheric window. Applied optics.

[21] Rex M (2020). PANGAEA.

[22] Haas C (2020). PANGAEA.

[23] Kanzow T (2020). PANGAEA.

[24] SENSOR. Device Information: iXBlue HYDRINS 1. *AWI Sensor Web* https://hdl.handle.net/10013/sensor.9ec5232e-9b30-492f-9741-63f056691cad (2021).

[25] Barber DB, Redding JD, McLain TW, Beard RW, Taylor CN (2006). Vision-based target geo-location using a fixed-wing miniature air vehicle. Journal of Intelligent and Robotic Systems.

[26] Andersen, O. B. & Knudsen, P. The DNSC08 mean sea surface and mean dynamic topography. *J. Geophys. Res*. **114**, 10.1029/2008JC005179 (2009).

[27] SENSOR. Device Information: Applanix AP60-Air. *AWI Sensor Web* https://hdl.handle.net/10013/sensor.a9fee346-91e7-4eed-9f2f-89f1368e53a0 (2021).

[28] Fischler MA, Bolles RC (1981). Random sample consensus: A paradigm for model fitting with applications to image analysis and automated cartography. Commun. ACM.

[29] Feldt, R. Blackboxoptim.jl. https://github.com/robertfeldt/BlackBoxOptim.jl (2021).

[30] Paturel, Y., Lacambre, J.-B., Patin, F. & Moynagh, C. Inertial navigation at high latitude: Trials and test results. In *OCEANS 2015-MTS/IEEE Washington*, 1–5, 10.23919/OCEANS.2015.7401904 (IEEE, 2015).

[31] Thielke L (2022). PANGAEA.

[32] Schmithüsen H, Raeke A, Kieser J (2021).

[33] Schmithüsen H, Schröter S, Wenzel J (2021).

[34] Schmithüsen H, Rohleder C, Hausen R (2021).

[35] Alhussein, M. & Haider, S. I. Simulation and analysis of uncooled microbolometer for serial readout architecture. *Journal of Sensors* **2016**, 10.1155/2016/9751056 (2016).

[36] Deutscher Wetterdienst (German Weather Service). Flight weather reports MOSAiC Leg 1, 2, and 3. *Personal communication* (2021).

[37] Nicolaus M (2022). Overview of the MOSAiC expedition: Snow and sea ice. Elem Sci Anth.

[38] Shupe MD (2022). Overview of the MOSAiC expedition: Atmosphere. Elem Sci Anth.

[39] Rabe B (2022). Overview of the MOSAiC expedition: Physical oceanography. Elem Sci Anth.

[40] Thielke L, Huntemann M (2022). Zenodo.

[41] Nixdorf U (2021). Zenodo.

